# The Cyclopædia of Practical Medicine. Parts I. and II

**Published:** 1832-04-01

**Authors:** 


The Cyclopædia of Practical Medicine. Edited by Drs. *Forbes*, *Tweedie*, and *Conolly*. Published in Monthly Parts, price Five Shillings. Part I. Abdomen—Aorta. Part II. Aorta—Auscultation. January and February, 1832.

It was high time that a Dictionary of Medicine should accompany a Dictionary of Surgery in this country. The success which crowned the latter work, though edited by an individual, might have reasonably prompted to a similar undertaking, in the wider field of physic; and Dr. Good, indeed, may be said to have made the attempt, though on a different plan, and with indifferent success. The London Medical Dictionary by Dr. Parr, of Exeter, was and is still, a very valuable performance, though greatly underrated and neglected in these times. It was evidently the Dictionnaire des Sciences Medicales that held forth the example, and furnished the model for the Cyclopædia now commenced in this country, and which promises to be of great importance to the profession. The editors may reap considerable advantages from the labours of their predecessors, Continental and British. They are cautioned by the bad example of the great French work, to avoid the introduction of unnecessary and extraneous articles, which increase the size but impair the strength of the performance—and they are warned by the Dictionary of Dr. Hooper, to aim at something more than a mere explanation of technical terms.* Every work, however, of the nature

* By the way, the explanation of technical terms might have been introduced, as they would have taken up very little space, and would thus save the necessity for a Medical Dictionary in addition to the Cyclopædia.

of this Cyclopædia, has certain attendant dangers, not easily avoided. Each article is, in fact, the *dissertation* of an individual on that particular subject—and therefore the public runs the risk of having the peculiar views or sentiments of the *individual*, instead of a judicious compendium of the actual knowledge which has been acquired by the whole profession. Here is the difficulty—here is the danger. Originality is more attractive for the first perusal; but it is not so permanently useful for subsequent reference as judicious compilation. This is the key to the great success of COOPER'S SURGICAL DICTIONARY—and if we look to antiquity, the same applies to the compilation of Celsus, in which the compiler hardly ever appears in his own person. These hints, we have no doubt, will be taken in good part, both by the principal editors and the collaborateurs, for we can assure them that we wish them success in their laudable and arduous undertaking.*

The first fasciculus, containing 112 pages of double columns, royal octavo, includes A B to A O, so that, if the work goes on in proportion, it will be of considerable, though we think not sufficient extent. We conceive that it should be on a large scale, so as to form a kind of library for the wide circle of practitioners, who cannot afford time or money for those works from which a Cyclopædia is derived. Dr. Forbes leads the van, in an able article on—

### EXPLORATION OF THE ABDOMEN.

Nothing can be more true than that medical men are, generally speaking, much too negligent in examining the external surface of the body while inquiring into internal diseases. This remark applies to the abdomen as well as to the thorax, where some diseases are strikingly expressed on the surface by change of configuration or alteration of natural movement. Thus, in chronic pleurisy, a single glance at the naked chest will shew immobility of one side, whether enlarged or contracted. In the abdomen, from the yielding parietes, the viscera are more easily examined, and enlargements or displacements detected.

Dr. Forbes has introduced three plates exhibiting regional plans of the trunk of the body, executed by Mr. Paxton of Oxford. We confess that we do not see their utility in a work of this kind. Under the heads of Inspection, Palpitation, Percussion, &c. of the Abdomen, Dr. F. makes many judicious observations, and offers some useful rules for the guidance of the young practitioner. Upon the whole, we suspect that "abdominal exploration" is somewhat too lengthy, as compared with other articles, and especially with the very next one to it—ABORTION. This last, from the talented pen of Dr. Robert Lee, occupies five columns, whereas the exploration, above-mentioned, claims NINETEEN. We shall introduce an interesting extract from Dr. Lee's article. After adverting to various general or constitutional causes of abortion, Dr. L. goes on to say—

"But by far the most frequent cause of abortion is in the product of con-

* We have reason to know that the views above-mentioned have been strongly enforced by the editors upon the attention of the collaborateurs, and we have no doubt that the result will be beneficial.—*Ed.*

ception itself; viz. in a diseased condition of the fœtus, or its involucra, by which it is deprived of life, and afterwards expelled from the uterus like a foreign body. The blighted ovum is thrown off from the parent, as fruit which has become withered is separated from the branch of the tree on which it has been produced. We have examined numerous ova which have been prematurely expelled, and in many of these, where no disease was obvious at first, some morbid state of the membranes, placenta, or embryo itself, has been detected, sufficient to account for the accident, wholly independent of any constitutional or local affection of the mother. Sometimes the chorion has been thickened, opaque, and extremely irregular, or lobulated, on its internal surface. The amnion, in some cases, has undergone similar changes, so that the healthy appearance of the involucra has been entirely lost. A collection of serum, or blood, has not unfrequently, also, taken place, between the chorion and amnion. The placenta, in some cases of abortion after the third month, has been hard, like cartilage, small and imperfectly formed, with calcareous particles deposited in its substance: in others, the placenta has been unusually large, and its vascular structure has been changed into a soft yellow fatty substance; or hydatids have been developed in its tissue. The umbilical cord, in these instances, has been remarkably slender, and the fœtus has appeared to perish for want of a proper supply of nourishment; and not from any defect in the organization of its internal parts.

The brain of the fœtus, or the thoracic or abdominal viscera, may all undergo various alterations of structure incompatible with life; and where the life of the fœtus is extinct, it becomes an extraneous body; expulsive efforts on the part of the uterus are usually soon set up, and abortion ensues as the necessary consequence. When the ovum is healthy, it adheres to the uterus with great force; but when diseased, the slightest shock to the mother, the most trifling mental affection, is sufficient to cause it to be expelled. Women have had the bones of the extremities fractured during pregnancy, and have suffered other grievous injuries, without miscarrying. A woman mentioned by Mauriceau escaped by a window from the third floor of her house when on fire, and in her fall to the ground fractured her arm, yet abortion did not follow. The case of a young woman with a narrow pelvis is related by Madame Lachapelle, who threw herself into a deep pit, and suffered injuries of which she subsequently died, yet the fœtus was not expelled.

All cases of abortion cannot, however, be referred to organic disease of the uterine organs, or of the embryo and its involucra; since it cannot be doubted that the process often arises from accidental detachment of the placenta, in consequence of an unusual determination of blood to the vessels of the uterus, or contraction of its parietes. The placenta adheres to the uterus by means of the deciduous membrane alone, which is directly applied to the openings of the uterine sinuses. If the impetus of the blood in these be increased by an excited state of the general circulation, or by irritation of the uterus itself, an unusual afflux of blood to these vessels will take place, and the placenta will be forced from its connexion with the uterus, more or less extensively, by the extravasation of blood from the openings of the uterine sinuses, between the placenta and uterus. If this takes place to a considerable extent, the process of gestation will be arrested, and in a longer or shorter period the ovum will be expelled. In plethoric women, or in those who menstruate copiously, very slight causes may give rise to a hemorrhagic effort in the uterine vessels, and to the extravasation of blood between the uterus and placenta, with the other consequences now described. A plethoric state of the uterine organs is most frequently met with in those who lead luxurious lives, who sleep in warm soft beds, and indulge to excess in animal enjoyments. This plethoric state of the uterus commonly gives rise to a sense of weight in the hypogastric region, or irregular pains of the uterus; but it sometimes happens that the blood suddenly bursts from the uterine vessels, and detaches the placenta, where there has existed no previous sign of unusual determination of blood to the parts.

Besides these causes, there are others which excite undue determination of blood to the uterine organs, as violent exercise, dancing, the use of the warm bath, the employment of acrid cathartics and emetics, spontaneous diarrhœa, the irritation of hemorrhoids, injurious pressure of the hypogastrium, and violent passions of the mind. Opening the membranes of the ovum and evacuating the liquor amnii certainly gives rise to premature expulsion of the contents of the gravid uterus." 11.

The symptoms which precede and accompany abortion are succinctly but clearly propounded, and the treatment is then explained.

"In premature expulsion of the ovum from organic disease of the uterine organs, or from alterations of structure in the embryo or its involucra, any plan of treatment is not only inefficacious, but even injurious, where the contractions of the uterus have been excited to throw off its morbid contents. Where the symptoms of abortion come on without any apparent cause, we have reason to fear that they arise from this state of the uterus or its contents. The treatment must be directed to the following points: first, to procure a complete separation of the ovum; secondly, to moderate the hemorrhage and pain which may accompany it.

By removing plethora, where there is general fulness and excitement, by venesection, and by calming the violence of the uterine contractions by sedatives, we shall often prevent a protracted discharge of blood; and by obtaining relaxation of the os and cervix uteri, favour the complete escape of the ovum. Where there are no signs of local or general plethora and excitement, blood-letting is contra-indicated.

In threatened abortion from congestion of blood, or unusual determination of this fluid to the uterus, with slight detachment of the placenta, and irregular uterine contractions, it is possible in some cases, by the prompt application of remedies, to arrest the mischief. The greatest mental tranquillity, and absolute rest in the horizontal posture, on a mattress or couch, with the body lightly covered, should be enjoined in all cases of threatened abortion of this description. If the patient is plethoric, and the pulse accelerated, blood is immediately to be detracted, in quantity proportioned to the urgency of the symptoms. Twelve or sixteen ounces should be taken from the arm, and, if necessary, the same quantity should again be taken after a time. Cold applications, and even ice, if it can be procured, should be applied over the pubis. A dose of laudanum, or liquor opii sedativus, is to be given, or a starch and laudanum clyster may be administered, to prevent or quiet the uterine contractions. The superacetate of lead is in these cases a valuable remedy. Two grains, combined with a quarter of a grain of opium, may be taken every three hours until the discharge of blood begins to abate.

As to the subsequent effect of abortion, it may be stated as a general fact, that, in a very large proportion of cases, it produces little or no injurious effect on the constitution of the mother. It is an accident of very frequent occurrence in all countries, and has often occurred without leaving any permanent injury. Where the process of expulsion has been protracted, and much blood has drained from the uterine vessels, a proportional injurious effect has been the consequence on the general health of the mother.

From what has now been stated respecting the causes and treatment of abortion, little requires to be said as to the management of women who are habitually liable to this accident. We are in possession of no means which can either prevent or remove the numerous organic diseases of the uterine organs, and of the embryo and its involucra. Much, however, may be done to avert the danger where it arises from plethora and irritation of the uterus alone, by obviating general fulness, and taking off the susceptibility to premature contraction of the uterine fibres, by rest, mild diet, and the occasional use of anodynes. Where there is much constitutional debility present, we must adopt all the means we possess for relieving the weakness, and more particularly the cold bath, and proper diet and exercise. Warm rooms and late hours are to be avoided." 13.

These extracts will shew that the article Abortion, though small in size, is pregnant with information.

Internal Abscess is well handled by Dr. Tweedie, one of the Editors. It occupies 14 columns, and is very creditable to that practical and industrious physician. It is quite insusceptible of analysis. We shall quote a single passage as a specimen.

"Abscess of the lung is a lesion very rarely met with, though from the frequent mention made of this pathological phenomenon by British as well as by some continental writers, we should be led to imagine it was a common termination of pulmonary inflammation. The opinion of Laennec on this point is very strongly expressed. It appears from his statement, that there is no organic lesion more rare than a collection of pus in the substance of the lung: and that, among several hundred dissections of persons who died of peripneumony, he had only met with a collection of pus in five or six instances, and in these the purulent deposites were neither extensive nor numerous in the same lung. In three cases only was the collection of pus of considerable extent, but in these there was no circumscribed cyst, the wall being formed by the pulmonary tissue which was much softened and disorganized. Laennec also states that he had been able to find only two similar cases on record, neither of which had the proper cyst of phlegmonous abscess.

Andral mentions the case of a new-born infant, whose lung contained several large abscesses. They had no resemblance whatever to tubercular excavation. (Vol. ii. p. 539, *Translation.*)

The kind of suppuration to which the substance of the lung is occasionally liable is purulent infiltration. When it occurs it is accompanied with hepatization, and appears to succeed to the most intense degree of inflammation. The consistence of the lung is at the same time much diminished, being so soft as to break down when handled. The pus appears in small detached points, so that when the lung is incised or squeezed, opaque yellow matter flows out. It may occupy an entire lobe, or only a small portion of it, and sometimes succeeds to pneumonia with great rapidity. Andral asserts that it has been found fully formed four days after the first symptoms of pneumonia had made their appearance.

The pulmonary parenchyma when infiltrated with pus presents a greyish ash colour, and, as the second stage of pneumonia has received the name of *red hepatization*, the former condition has been distinguished by the term *grey hepatization*. When the lung, in this state, is pressed, the purulent fluid exudes in greater or less quantity, and when it is squeezed out, the lung frequently reassumes the red colour and hepatized appearance of the second stage; thus proving, as Andral states, that the *grey* hepatization differs only from the *red* in having its structure infiltrated with pus.

The process of softening of tubercles presents another variety of purulent formation in the substance of the lung. These bodies, which vary in size, are in their first stage semi-transparent, and of a greyish colour; in some cases almost colourless and transparent. In this state they are productive of little inconvenience; and hence individuals, with pulmonary tubercles, often enjoy uninterrupted health for years. Sometimes a single tubercle has been found, but more generally they are numerous, and situated in the upper part of the lung. In some cases they are superficial, in others deep-seated. The tendency of these bodies is to soften, and to form a cavity in the portion of the lung where they are situated. The softening generally commences in the centre, and gradually increases towards the circumference, until the whole tubercle is converted into a fluid mass. One or more of the bronchial tubes become perforated, through which the tubercular matter is evacuated. A tubercular cavity is thus left, the interior of which is traversed by bands of pulmonary tissue, covered by tubercular matter, and the smaller branches of blood-vessels, which are generally obliterated and transformed into impermeable cords. In some instances, however, these vessels continue open, and, by their erosion or rupture, occasion hæmoptysis. The larger blood-vessels are pressed towards the side of the excavation, lining, as it were, its internal surface. The bronchial ramifications undergo similar pressure; but, according to Laennec, they appear to be rather enveloped than pressed aside by the tubercular matter, the pressure apparently soon obliterating their canal, as they are scarcely ever to be detected in the morbid structure of the lung. He moreover thinks they must have originally traversed the portion of lung occupied by the tubercles, as, in even the smallest excavation, one or more bronchial tubes are found opening into it. These tubes appear not to open sideways, but are cut directly across on a line with the internal surface of the excavation, while, from their direction, it is evident that they originally crossed the excavation. The boundary of these tubercular cavities is formed by the parenchymatous tissue of the lung, which has become more or less indurated or infiltrated with tubercular matter. The internal surface is lined by a false membrane, which is composed of concrete pus, and is so soft as to be easily detached by the scalpel.

These cavities vary in number and size; sometimes there is only one cavity, in others there are several, which either remain isolated, or communicate together by fistulous openings, varying in extent from such as will scarcely contain a filbert, to those which occupy a considerable portion of one lung.

With regard to the possibility of the cicatrization, or healing of tubercular excavations, it would appear, from the minute and distinct researches of Laennec, that this process does take place, though in a very small proportion of cases." 17.

Dr. Marshall Hall has given a short but interesting article on ABSTINENCE, which he treats of under three heads—its curative effects—its morbid effects, when employed as a remedy—and its morbid effects in famine, &c. We shall make room for an extract from this article.

"Abstinence is also a very valuable remedy in many of the more chronic forms of disease; a state of extreme inanition of the stomach not only enfeebles the circulation, but acts most powerfully upon the absorbent system. With this view it has been proposed to institute a system of rigid abstinence in cancer: it might probably be more successful in some kinds of dropsy.

An obvious application of abstinence, as a remedy, is that made in cases of disorder or disease of the stomach itself. These affections are so constantly the effects of improper food, that, to change the diet is obviously but to remove the most usual cause. To withdraw food nearly altogether for a time would, doubtless, be to employ an actual and powerful remedy. This is so evident, and yet the facts in illustration are so few in number, that we cannot but recommend the subject for future inquiry. If a joint be morbidly affected, we enjoin the most absolute repose. The value of a well-regulated but rigorous abstinence in cases of disorder or disease of the stomach itself, and of some of the chylopoietic viscera, especially the liver, would, doubtless, be equally great. This subject has been well touched upon by Dr. J. Johnson; and the writer of this article can add his testimony to the beneficial effects of abstinence in the cure of these diseases.

It is impossible to refer to the subject of the use of abstinence as a remedy in disorders or diseases of the stomach, without recalling to our minds the services which M. Broussais has rendered to this department of medicine: the *diète absolue*, or extreme abstinence, recommended in gastritis by that author, is, assuredly, a far more natural and appropriate remedy, than the mistaken administration of drastic purges, too much employed in this country.

The next object in enjoining abstinence, is to reduce the mass of solids and of fluids. Abstinence is, therefore, the most direct remedy for plethora. This point has been ably treated of by Dr. Barlow, of Bath, in his Treatise on the Bath Waters. See the Article ANTIPHLOGISTIC REGIMEN.

The case which next presses itself upon our notice, in regard to abstinence as a remedy, is that of disease, or tendency to disease, within the head. The immediate threatening of apoplexy must be treated by active blood-letting; but the remedy for the permanent disposition to this disease is the most strict abstinence. The most rigorous system of weighed or measured portions of the least nutritious and least stimulant kinds of food is to be enforced.

We must now add a few words upon the celebrated use of abstinence in diseases of the heart and arteries, in the hands of Valsalva, as described by Morgagni, (lib. ii. ep. xvii. § 30,) from the account given to him by Stancarius. Sufficient blood having been taken, Valsalva ordered the food and drink to be daily diminished, until it was reduced to half a pint of gruel in the morning, and less than half that quantity in the evening; to this water alone was added, and that in a certain measure only, medicated by the addition of quince, &c. When the patient was, in this manner, emaciated, and so debilitated that he could not raise his hand from the bed, the diet was slowly and moderately increased, so as just to maintain power enough for raising the body. In this manner these formidable diseases were cured. It is confessed, (§ 31,) that some thought the cure of Valsalva worse than their aneurism. But it was argued that that which was not done early in the disease, with the hope of cure, might become necessary during its progress, without such hope, from the incapacity for swallowing." 21.

The author makes many judicious observations on the injurious effects of abstinence, too long or unnecessarily enjoined. After referring to memoirs which we have published in this Journal, Dr. Hall conveys his own opinion on the morbid effects of inordinate abstinence, in the following passage.

"The first part of the series of the effects of abstinence consists in simple debility and emaciation. The countenance becomes pale, and the expression languid; the muscles of voluntary motion become thinner and feebler; the pulse feebler and smaller. The second part of the series is different. The pulse is augmented in frequency; there is often palpitation of the heart, alternately with syncope; and there is pain of the head, or delirium; symptoms which might be mistaken, by the inexperienced or unwary, for those of increased action and power, whereas they are but the feeble flashes or glimmerings of a light ready to become extinct.

Such a transition in the effects of abstinence is frequently the consequence of an undue administration of this remedy; still more frequently, however, it arises from its inappropriate application. When cases of irritation are mistaken for those of inflammation, the morbid effects of abstinence are particularly apt to occur. The former disease neither requires nor bears the remedy; whilst, in inflammation, it is both essential to the cure of the disease and well sustained by the patient. This is a point which requires to be well investigated. The ancients, and especially Celsus, speak of a principle of encouragement in the sick which they expressed in these words—*difficulter ferre morbum*, (lib. iii. § 3.) There is another principle not less important—*difficulter ferre* REMEDIA. It would be difficult to induce the morbid effects of abstinence when there is a tendency to apoplexy; but it would be very easy to do so if palpitation, or some other symptom of gastric irritation, were mistaken for disease of the heart, or for inflammation." 21.

Under the head of famine, &c. Dr. Hall has collected much curious and interesting information; but it is unnecessary to dwell on the subject in this place.

ACHOR and ACNE are very well treated of by Dr. Todd, of Brighton. These two articles occupy 18 columns. Achor is considered by Dr. T. to be a cutaneous affection peculiar to children, constituting the elementary form of some porriginous diseases. It seems to be connected with a plethoric state of body and chylopoietic derangement, being most effectually relieved by mild alterative doses of the hyd. cum cretâ, followed by rhubarb and magnesia. Acne occupies 17 out of the 18 columns, and, consequently, is minutely investigated in all its forms and stages. We shall introduce an extract from acne rosacea, or carbuncled face, as a specimen.

"The nose is, commonly, the first seat of this affection. In persons predisposed, who are generally of the middle age, after any exciting cause, as a full meal, heating drinks, or indigestible substances, the extremity of the nose becomes of a deep red colour, more or less intense, which at first gradually subsides with the removal of the exciting cause, but, at length, by repetition, grows to be habitual. In this red shining appearance of the nose, some elevated points of a brighter red colour, sometimes distinct, sometimes in groups, are afterwards observed. These points enlarge, becoming pustules, which suppurate at their summits; but the suppurative process is always imperfectly established, forming seldom more than a small white acuminated point on the apex of the pustule, which makes a striking contrast with the dark damask red colour of the pustule, an appearance which is well represented in Alibert's XXI. plate. This white point of the pustule bursts and forms a thin white scab, which, detaching itself, leaves beneath it a hard phymatous tubercle. These pustules succeed each other, and in this manner the disease perpetuates itself. Sometimes the disease is confined exclusively to the nose, which, from the repetition of this morbid process, and the successive formation of tubercles, increases very considerably in size, being covered with knobs and asperities, whilst the blood-vessels of the surface of the skin are seen enlarged, and the small veins appearing varicosed, have the appearance of bluish lines, strongly contrasted with the general red colour of the surface; or the intermediate skin is striated with reticulations of enlarged cutaneous veins, resembling an injected membrane, an appearance which is correctly delineated in Bateman's LXIV. plate. M. Alibert thinks he has observed, that the right side of the nose is more liable to be affected with this species of acne than the left, a circumstance which he connects with the influence of the state of the liver upon this disease. More generally the size of the nose is not increased, but its form only altered; and the disease extends itself to the cheeks, forehead and chin, so as sometimes to cover the whole face. The red colour of the skin, which is always more remarkable after dinner, or in the evening, than in the morning, is not any where equal in degree, but is always more so in the seat of the pustules. The disease may cease, and return in different degrees of intensity; but after it has continued for some time, the surface of the skin becomes uneven and rough, and if the disease should even disappear, the skin never entirely recovers its natural state.

In general, this eruption produces little discomfort of feeling in proportion to its deformity, seldom more than a slight momentary itching: but some persons have the face in a very irritable state, with a sensation of heat and burning, being frequently obliged to bathe the face in cold or tepid water for relief. After eating, drinking, or any moderate exercise, they feel a sudden glow of heat in the face; but it is chiefly when they approach the fire that they suffer most; it produces in them a sensation of pungent heat, or of burning and itching." 30.

The complaint is supposed by Dr. T. to generally depend on a state of abdominal plethora, or congestion of the liver, rather than upon irritation in the mucous membrane of the primæ viæ. Regulation of diet and regimen is of more consequence than medicine.

"If much local inflammation be present with general plethora, a moderate venesection may render great service. If the eruption is connected with the suppression of any accustomed evacuation, the propriety of endeavouring to restore it naturally suggests itself; if the menstrual function is laborious, imperfect, or irregular, the proper modification of the method of cure readily occurs; if a congestive state of the liver is present, or when the liver is more severely affected, mild alterative remedies—as Plummer's pill, taraxacum, and sulphurous mineral waters, as those of Harrogate, are applicable. These last are of known efficacy, and are much to be preferred to the saline mineral waters. In all cases an open state of the bowels is of importance, but much purging is generally detrimental. We have great doubt whether any constitutional remedy has a specific action upon the local disease. In France and Germany the dulcamara and viola tricolor have been much used; but we think that, united with the general treatment, more benefit is derived from the use of the liquor potassæ taken a few hours after each meal." 31.

This is certainly a neatly executed article.

Acupuncture is from the pen of Dr. Elliotson, and, of course, is interesting. That acupuncture gives *temporary* relief in rheumatic and neuralgic affections unaccompanied by inflammation, we believe, because we have witnessed this relief; but that it has ever effected a cure, we do not believe; because, in every instance that came under our observation, and in many cases reported to us by others, the disease returned. Still, as a temporary refuge from pain, it is a remedy not to be despised, under the limitations above-mentioned. We agree with Dr. E. that, "the most obvious purpose of this operation is to allow the escape of the fluid of œdema or anasarca through the skin, or of the blood when superficially accumulated."

Age, by Dr. P. M. Roget, occupies upwards of 20 columns, and as might be anticipated, is executed with talent and erudition. The following brief and rapid sketch is emblematic of the fleeting shadow of man's existence, which passes like a cloud—is dissolved in air—never more to be seen!

"As youth is the state of transition from infancy to maturity, so age is the state of transition from maturity to decay. In the early periods of life, all the powers of the system are directed to the building up of the frame, and of the different organs; to their extension, consolidation, and perfection; and to their adaptation to the performance of their several functions. The exertions made for the attainment of these objects are great, and commensurate with the magnitude and importance of the design; and they give rise to a rapid and varied succession of changes. An abundant store of materials is wanted for these operations; and although the consumption and renovation of these materials be considerable, yet the supply much exceeds the loss; and the body, accordingly, continues to augment in bulk. In course of time, these opposite processes of reparation and decay approach nearer to an equality, and, at length, are exactly balanced. The parts then cease to grow; the system has reached its state of maturity; and the object of the vital powers and functions is now to maintain it in a uniform condition of health and vigour, qualified for the exercise of all its physical and mental faculties. It cannot but excite our admiration to contemplate the accuracy of the adjustments by which these objects are so perfectly accomplished, and that equilibrium preserved, with such wonderful constancy, for so long a period of years.

But at length there comes a season when the balance, hitherto so evenly kept, begins to incline; the powers of the system are less equal to the demands made upon them; a diminution of energy becomes sensible; and the waste of the body exceeds the supply. Yet nature is far from abandoning her work: new arrangements are made, and new provisions resorted to for accommodating the system to these changes. In proportion as the supply of materials for repairing the waste of the organs becomes less abundant, a more strict economy is adopted; the resources of the system are husbanded with greater care; and the functions thus appear to go on for a considerable period without any material or very manifest alteration. Yet all this time the changes which are going on, though insidious, are no less real. Old age steals upon us by slow and imperceptible degrees, which, even when obvious to others, are often unknown to ourselves. Nature, when the system is entrusted wholly to her laws, thus kindly smooths the path along which we descend the vale of life, and conducts us by easy stages to our destined place of repose. But the number of those who thus gently glide along the stream of years is small indeed, compared with those whose declining age is withered by infirmities or embittered by disease. The 'Age that melts in unperceived decay' is rarely met with amidst the numerous and diversified causes of premature decrepitude, to which man, in his civilized condition, is obnoxious." 35.

We must indulge ourselves and our readers with one other abstract from this highly interesting article.

"The mind, as well as the body, is wasted by time. The first indication of diminished vigour in the intellectual faculties is usually the decay of the memory. The power of recollection, which is immediately dependent upon that of association, appears to have a closer relation to the physical condition of the *sensorium*, than any other of the mental faculties: for we often observe a failure of memory, while the judgement continues unimpaired. This loss of power is chiefly felt in the case of new associations. Thus recent events are recalled with much greater difficulty than old ones; and new habits can hardly ever be contracted. The earliest notice that is given of this partial decline of the faculties is generally in the forgetfulness of the names of persons. When carried somewhat farther, the names of things are with difficulty recollected. The mind loses that command of language which it formerly possessed; hence the tardiness of speech, and heaviness of expression which characterize the conversation of so many persons of advanced age. The garrulity of old persons is also frequently a consequence of the deficiency of memory, which effaces the recollection of what has just been said, and leads to continual reiteration of the same ideas.

Not only are the bodily feelings impaired by age; the mental sensibility is also blunted, in at least an equal degree, in all that relates to present or recent impressions. Yet it has often been remarked that old persons feel acutely the loss of former friends and companions. How often do we not witness the survivor of an aged couple soon follow his partner to the tomb. The failure of the sight, of the hearing, the senses which connect us most largely with the external world, contribute much to the diminished exercise of the intellect, by abstracting the occasions for exertion; and we well know that, without exercise, the intellectual as well as the bodily powers stagnate and become torpid. To this cause are often added impediments to bodily exertion arising from rigidity of the membranes, stiffness of the joints, debility of muscles, and impaired nervous energy. The tottering steps and tardy movements of the infirm old man can be accompanied with none of the enjoyment which attends the exertions of limbs animated by the elastic spring of youth. If, under these circumstances, he should unfortunatly be deprived of the resources of mental cultivation, can we wonder that he is driven for refuge to the enjoyment of those senses of taste and smell that yet remain; and that he devotes himself to the cultivation of the pleasures of the table, and the artificial excitation of spirituous liquors? Yet even here nature imposes certain limits, beyond which the votaries of luxury are forbidden to pass.

'Time hovers o'er, impatient to destroy,
And closes all the avenues of joy.
In vain their gifts the bounteous seasons pour,
The fruit autumnal, and the vernal shower;
With listless eyes the dotard views the store,
He views, and wonders that they please no more.
Now pall the tasteless meats and joyless wines,
And luxury with sighs her slave resigns.'

Need we pursue this 'strange eventful history' to the last melancholy chapter of man's existence, and contemplate the wreck of those exalted faculties which ennoble his nature, and of which the deprivation lowers his condition far beneath that of the beasts of the field? Need we dwell upon the sickening spectacle of 'second childishness and mere oblivion;' and disclose those mournful contrarieties of our nature, that drew forth the exclamation from the poet—

'In life's last scene what prodigies surprise?
Tears of the brave and follies of the wise.
From Marlborough's eyes the streams of dotage flow,
And Swift expires a driveller and a show.'

Let us rather draw a veil on this humiliating picture of the frailties incident to humanity, and which forcibly remind us of what

'We shun to know,
That life protracted, is protracted woe.'" 42.

Dr. Roget has speculated a little by going in search of some single principle that may account for the various stages of transition from infancy to senectitude, or rather from the embryo to the death of the individual. We shall give this speculation in his own words.

"Admitting that the increasing density of the cellular substance is the natural consequence of the diminished force of circulation, aided, perhaps, by the increased, or, at least, undiminished power of absorption, may we not advance a step farther, and ascribe the diminution of the force of circulation to the gradual loss of muscular power arising from a decline in the energies of the nervous system? If this be a legitimate inference, then this declension of nervous power, which takes place with more or less rapidity as we advance in life, appears to be the general principle we were in quest of; that is, the ultimate fact to which all others are subordinate. Appearances, then, warrant the hypothesis that a certain stock of vital force is imparted to the embryo at its first formation, as a provision for carrying it through its destined career of existence. In every action of the system a portion of this power is expended; and the greater the expenditure, the less must there be remaining, till at length, the whole being consumed, all movements cease, like those of a watch which has run down, and of which the main spring has ceased to act." 42.

To this we would reply, that there is just as good ground for believing that the "nervous power" is regenerated or reproduced in the brain, as that the blood is replenished daily from the chyle—and all parts of the system daily recruited from the blood. If there was a certain stock of nervous power conferred on the embryo at its first formation, without the power of recruiting that stock, we cannot well conceive the *increasing power* till a certain period, and then its decline. We fear that we know little more than this, that the animal machine comes to maturity of action by powers implanted in it by the hand of the Creator; but that, after a certain period, it begins to feel the wear and tear of time, and at length breaks down like an old hackney-coach in the streets, rather than like a watch, whose main-spring has ceased to act, but which may be easily wound up again by a proper key.

The diseases which play their part in the decline of life are lightly touched upon by Dr. Roget, and the whole article is extremely creditable to that enlightened physician.

CHANGE OF AIR, by Dr. J. Clark, occupies half a dozen of columns, and, like all that comes from the pen of Dr. C., is sensible and judicious.

In the article ALOPECIA, by Dr. Todd, there is shown considerable research. The following extract will shew how this article is treated.

"The immediate cause of the falling of the hairs is unquestionably a diseased state of the follicles which nourish and support their bulbs, having the same relation to them as the capsule or membrane which surrounds the roots of the teeth, (for the process of the formation of the teeth and the hair is perfectly the same); but the precise nature of the diseased state has not been determined. It would seem to depend sometimes upon inflammation of the follicles, sometimes upon their ulceration, sometimes upon a temporary deficient action, and sometimes upon atrophy or death of the follicles. In the body of a man who had become almost entirely bald in consequence of a putrid fever, of which he died, Bichat observed all the pilous follicles in their natural state, and small hairs shooting forwards from their bottom; but he remarks that, before the fall of the hair in aged people, the cavity of the bulbs of the hairs gradually diminishes, and the follicles, which contain the bulbs, at last disappear. The destruction of the pilous follicles may, however, be caused by pressure, by friction, and by other causes. Thus it has been observed to be produced by the pressure of certain subcutaneous tumours.

Alopecia may be a purely *local* and *idiopathic* disease; the affection originating in the follicles themselves. This happens when it arises from external causes, as from the application of quick lime, or other depilatories; from the fumes of quicksilver, as was observed by Forestus in goldsmiths; from exposure of the head to the rays of the sun; from frequent pressure of weights upon the head; and from friction of any hairy surface by the garments or otherwise. Or it may be *local* and *consecutive*, as when the follicles are injured by becoming involved in the inflammation, ulceration, or other morbid process of any adjacent cutaneous disease, as happens in porrigo, impetigo, variola, eczema, elephantiasis, and several others.

Alopecia may also be *secondary* and *symptomatic*, a consequence of general debility and constitutional exhaustion, and hence it attends the convalescence of febrile diseases, and the puerperal state; hence, also, it is a common symptom of the advanced stage of phthisis, of diabetes, and of most cachectic diseases: thus justifying the prudence of the Romans, who estimated slaves affected with alopecia at the lowest price. Hence it is also observed in the nervous debility which follows excessive venereal indulgences or seminal emissions, and has been known to be produced by painful and distressing headachs; by long-continued and intense study; by the depressing passions, as fear; by cares, disappointments, and anxiety. Of this kind was evidently that singular case related by Ravator, of a person who, after a violent commotion, was attacked with amaurosis of the right eye, and all the hairs of the same side of whose body lost their colour, and fell from the eye-brows and eye-lashes as well as from the head. Of the same nature, also, was, in all probability, the remarkable case of M. le Chevalier d'Epernay,* who after an assiduous application for the space of four months, without any previous symptom of disease, lost his beard, his eye-lashes, his eye-brows, and, in short, all the hair of his head and body.

Alopecia may be a *sympathic* affection, not a symptom of a constitutional disease, but caused by a disease or disordered state of some other organ or system of organs. The most common form of this description which has come under our observation is that which proceeds from chronic inflammation of the mucous membrane of the stomach, giving rise to a particular form of dyspepsia, which has, for this reason, been called inflammatory." 50.

Dr. Conolly, one of the editors, first presents himself as writer of the article Alteratives. Seven columns are dedicated to a consideration of this somewhat vague term, which is now rather receding from the nomenclature of remedial agents. It is well drawn up by the accomplished author, as the following specimen will shew.

"Mercury, in all its various forms, is one of the medicines most commonly employed as alterative; and the great influence it exerts on the whole economy, over all the secretions and excretions and over the nervous system itself, constitute it an alterative, when prudently given, of a most efficacious kind. Even in certain states of fever, mercury has been employed with success for the restoration of the secretions, and, therefore, it may be said, as an alterative. In chronic inflammations, although here, perhaps, the term alterative may be objected to, small doses of the pilula hydrargyri or of calomel are often considered highly serviceable: no cases are more frequent than these, and in none is the practitioner more in need of some means of checking or altering actions, which, although neither violent nor immediately dangerous, are silently effecting structural changes and irreparable mischief. It would certainly appear that, for this purpose, the majority of practitioners rely on the efficacy of mercury, often in combination with opium. In certain instances, of which chronic laryngitis may be cited as an example, as well as in the instances of new formations, even of a malignant character, the addition of a medicine possessing narcotic properties may be useful on the principle of allaying the disturbance of the nervous system, a disturbance but little regarded or acknowledged in such cases, but probably intimately connected with the primary functional disorder in which all morbid changes of structure, and even inflammation itself, must commence. Most persons of experience in medicine have met with examples of chronic disorders of a troublesome rather than of a dangerous nature, which have been ameliorated, or entirely relieved, by the persevering use of some of the forms of mercury; although the medicine may have been given at first without any other reason than that it afforded a chance of benefit. Even irritable states of the bronchial and intestinal mucous membrane certainly sometimes give way under this treatment; but the application of it requires that caution of which nothing but observation can teach the value. The advantage obtained in such cases, and in others not unfrequently met with, from the apparently indiscriminate employment of calomel, may eventually be found to depend upon some general law, which has not yet been explained; or simply, as we believe John Hunter thought, on one kind of irritation superseding another, and banishing it from the system. No medicine is so commonly given in disease of the mesenteric glands as calomel; it is by no means rarely administered in scrofula; notwithstanding the general opinion

* Gazette François, Feb. 23d, 1763.

of the unfavourable influence of mercury on the scrofulous constitution; and notwithstanding the common accompaniment with mesenteric disease of a state of intestinal irritation or of chronic inflammation. In almost every varied disturbance of the liver, mercury is one of the first medicines to which many practitioners have recourse, and in the form of the pilula hydrargyri it has been recommended in many disorders of the digestive functions. A practice so common must have been supported by many cases in which it was found useful, although the principle on which the medicine acts, if it be not that of suspending morbid actions, is, in some of the cases, not very easily imagined. In the case in which acute inflammation of membranous parts is checked by the employment of calomel; or depositions, the consequences of such inflammation, are removed, of which iritis may be mentioned as presenting a striking illustration; this medicine is given to produce a precise effect, which experience has shewn to arise from its use. In the chronic forms of indigestion, its operation on the secretions seems to explain the great advantage often arising from it. In the other cases, cases of mere irritation, or cases in which there is a disposition to new formations not ascribable to inflammation, the same medicine is given, often with the same good effects; but the actions which are then interrupted being less understood, the medicine is only called an alterative." 53.

AMAUROSIS, by Dr. Jacob, of Dublin, exhibits an excellent specimen of careful compilation, under the guidance of sound judgment and extensive knowledge in the person of the compiler. All the best ophthalmologic writers are laid under contribution by Dr. Jacob, while no small portion of original information is interwoven by the author into the tissue of the article. A quotation from this dissertation would be like a specimen of St. Peter's by means of a block of Tiburtine stone.

AMENORRHŒA is from the pen of Dr. Locock, and is concise, but much to the purpose. The following brief extract will suffice as a specimen.

"The causes of this disease may be shortly stated, as all those which depress the vital powers, viz. a previously delicate and unhealthy childhood, insufficient or improper food, want of pure air and exercise, too close a confinement to study in schools, or to labour in crowded manufactories, the depressing passions, and, in particular, according to many, hope deferred, and disappointed sexual feelings.

In treating the disease, the amenorrhœa must at first be considered as only one of the train of symptoms of disorder of the general health. It is advisable to begin with an active purgative, which will often bring away a large collection of highly offensive motions, with manifest relief to the patient. Small doses of blue pill may be afterwards occasionally repeated, and purgatives of a warm and stimulating character taken every morning, combined with a small quantity of some bitter extract or infusion, until the tongue appears cleaner, and the secretions from the bowels are more healthy. A more decided tonic of the vegetable class, along with myrrh, rhubarb, or aloes, and ammonia, will gradually prepare the stomach for the metallic tonics, and above all others for that medicine most useful in these cases, namely iron, which, in one form or another, may be nearly always taken with benefit in a torpid condition of the venous system. Upon the whole, perhaps, the Griffiths's mixture (mistura ferri composita of the Pharmacopœia) is the most serviceable of the artificial preparations of iron. At the same time the bowels must be kept fairly opened with the above-mentioned purgatives, those containing aloes being preferable. The diet must be, at first, light and easily digestible; and, as the stomach is prepared for an improved and more nourishing food, wine, meat, and eggs may be taken. Gentle exercise in a carriage or on horseback, particularly the latter, with sea-bathing or the showerbath, may be ventured upon cautiously as the strength improves. A pure air is very desirable, and on that account, when the patient has a little advanced, nothing is more efficacious than a residence at Tunbridge-Wells, or some other places where chalybeate springs abound, combining the advantages of change of scene, a salubrious atmosphere, amusement to the mind, and the internal use of the mineral water." 69.

It often happens that the menstrual secretion does not return with restored general health; and then we must have recourse to emmenagogues. Among these are aloes and black hellebore, lytta and savine, myrrh and iron, galvanism and semicupia. Dr. Locock has found the essential oil of savine beneficial; and we have seen the extractum sabinæ excite menstruation when nothing else would. The injection of liquor ammonia with milk into the vagina, has sometimes succeeded. Dr. Coindet, of Geneva, considers iodine as the most powerful emmenagogue. Dr. Locock has exhibited the ergot of rye, with more success, in doses of ten grains twice a day. In irritable habits this medicine should be administered with some caution, as it occasionally excites violent spasms. There is a great deal of information crowded into a small space in this article of Dr. Locock's.

Anasarca undergoes a considerable extent of investigation, by Dr. Darwell, who draws his information from various sources, but chiefly from Abercrombie, Blackall, Wells, and Crampton. Under the head of Local Anasarca, Dr. D. states it as his opinion that the investigations of Dr. Davis and others have set the pathology of phlegmatia dolens at rest. We are of a very different opinion.

Angina Pectoris is very ably portrayed by Dr. Forbes. We think he has expended some unnecessary labour and erudition on the etymology and synonymy of this curious disease; and there is no doubt but that the whole article is swelled far beyond its *relative* proportion to others of equal or greater importance. We say *relative*, for we do not think that Dr. Forbes has said a word too much respecting the etiology, pathology, or treatment of the disease. We agree with Dr. Forbes in believing the heart itself to be the seat of the pain, during the paroxysm—that the pain is of the neuralgic or spasmodic character—that it often occurs without any organic disease—and that the structural change, when existing, can only be considered in the light of a predisposing or exciting cause of the paroxysm. The following classification is adopted by Dr. Forbes.

"I—*Organic Angina.*
1. Pure, or idiopathic.
2. Complex, or sympathetic.
II.—*Functional Angina.*
1. Pure, or idiopathic.
2. Complex, or sympathetic.

1. The cases that come under the first subdivision of organic angina are few in number. They are those in which the anginous paroxysms seem to be the direct consequence of organic disease of the heart occurring in persons otherwise healthy. Cases of this kind are seldom very well marked, the anginous symptoms being either feebly manifested, or overpowered by the greater intensity of the more ordinary symptoms of heart disease. These may be considered, in one respect, as the worst cases of angina, inasmuch as they hold out little prospect of cure or even of alleviation. Our influence over diseases of the heart is very slight, except they are partly the effect of some other disorder of a more curable kind.

2. Under the next subdivision of organic angina, we would include the greater number of the best marked and more severe cases of this disease. In these, along with the organic affection of the heart or vessels, or both, (probably not very great, or, at least, marked rather by the paroxysm of angina than by the general symptoms of diseased heart,) we have some obvious general disorder of the system. In cases of this kind, the organic disease of the heart and aorta seems often to be a consequence of the co-existing disorder; if not a consequence, it is always greatly aggravated by its presence; and hence the most successful medical treatment of the angina is that which has direct reference to the concomitant disorder.

Among the various structural affections formerly detailed as constituting the essential or organic character of these two classes of cases, different authors at different times, have been anxious to select some one lesion as the exclusive cause of the disease. The principal of these have been,—ossification of the coronary arteries, ossification and dilatation of the aorta, accumulation of fat in or around the heart, &c. To these may be added, with equal propriety, several other morbid states of the heart, particularly—softening of the muscular substance of the heart, dilatation of one or more of the cavities, &c. It will be at once admitted as a necessary consequence of the result of the dissections given above, that no one of these lesions is entitled to be considered as exclusively the cause of the paroxysm of angina; and that some of those which have been most generally believed to be such, are equalled or exceeded in point of frequency by others. Thus we see that the ossification of the coronary arteries, formerly considered by so many as the chief or sole cause of the paroxysm, is only of the same frequency of occurrence as disease of the valves, while disease of the aorta is much more frequent than either. But we think it probable that much slighter deviations from the normal condition of the organs of circulation than any above noticed constitute quite as frequent causes of this disease; more particularly of the milder cases. In a certain proportion of this class of cases it is not always possible to detect, during life, the existence of any organic lesion, much less the precise lesion; and from the same cause, (its slightness,) it is frequently overlooked in the examination after death. In a considerable number of such cases, however, the nature of the morbid deviation is discoverable both during life and after death. The most common of these slighter deviations from healthy structure are, a thin and slightly dilated state of the ventricles, and a want of tone in the muscular fibre.

3. We consider cases of the kind just mentioned, in which the organic deviation is so slight as to be hardly discoverable, as constituting the greater number of those usually viewed by practitioners as examples of pure functional or nervous angina. It is obvious that in extreme strictness of language they are not entitled to this name; yet if the deviation is only so slight as to constitute mere feebleness, (and it is often nothing more,) they are probably as well entitled to the name as most other diseases commonly denominated nervous. But it must be admitted that in persons possessing the best proportioned hearts, and in which no deviation whatever from the normal structure can be detected either during life or after death, there may and do occur paroxysms of angina. The proportion of such cases is however very small under any circumstances in a state of uncomplication with other diseases; and we look upon them rather as of possible occurrence than as having certainly met with them in practice. Conjoined, however, with some other disorder, as in the next class of cases, we conceive they are by no means rare.

4. Under the head of complex or sympathetic functional angina, we must comprehend a large class of cases; and, for the reasons stated in the last paragraph, although not strictly philosophical, we would, for practical purposes, include under the present division all the cases of nervous angina complicated with other diseases, whether the organs of the circulation are perfectly sound and well proportioned, or only deviating in a very slight degree from this state of integrity. Under this head are comprehended a very considerable proportion of the cases met with in practice, and not a few of those which present symptoms of the greatest severity in the paroxysm." 88.

The phenomena claiming the name of angina pectoris, are frequently complicated with dyspepsia, which, both in its earliest and latest stages, aggravates almost every other complaint. Plethora, obesity, gout, and rheumatism are frequently connected etiologically with the disease under consideration.

Some diversity of treatment is necessary according to the constitution of the patient and the nature of the complaint. In the robust and plethoric, venesection sometimes relieves the paroxysm. Under other and different circumstances, however, we must have recourse to a different class of remedies. Opiates are less effectual than might be expected, in severe cases. A paroxysm rarely occurs without a previous accumulation of gas in the stomach, which, indeed, often appears to bring on the attack. The extrication of this gas is of the greatest importance. Dr. F. touches on the subject of dyspepsy, as a frequent cause or complication of angina pectoris, in the following terms.

"Of the treatment of simple or primary dyspepsia, as co-existing with angina, we shall only here remark, that much more is to be effected by a rational system of diet and regimen, both of body and mind, than by medicines ; and that, as a general rule, the cautious, cooling, and macerating treatment of our continental neighbours, will be found more successful than the endless ingestion of bitters and drastic purges, so much practised in this country. In many cases, no doubt, the stomach is simply debilitated, and requires tonics ; but it is much more frequently irritated or inflamed, and requires soothing and depletion. The prevailing and most injurious error among many practitioners, is, apparently, to overlook the fact, that the stomach or intestinal canal can be inflamed without pain or the more common external marks of febrile action ; or, being so, that they can be injured by the stimulus of purging. But it is in the complex constitutional disorder, which may be termed *secondary dyspepsia,* that these observations are most applicable. It is to be feared that the true nature of this disorder is not well understood by the generality of practitioners. It is one of those diseases which may often be said to be the *opprobria medicorum* in the worst possible sense ; since it is not seldom caused, and is very frequently fixed and perpetuated, by injudicious treatment. In no other disease is the over-active, or, as our neighbours term it, *heroic* mode of practice, so prevalent in England, productive of such evil effects ; and in none is that mild, simple, yet comprehensive system of treatment, which embraces the whole of the disordered organs and functions, and rather prompts nature to act rightly than supersedes her agency, so strikingly beneficial." 92.

Under the head of ANODYNES, Dr. Whiting has given us a short, but interesting disquisition on the nature and causes of pain, for which the anodyne is prescribed. The term indeed is very comprehensive. Almost all diseases are accompanied by pain—and consequently every thing that removes the cause of pain is an anodyne. Nay, what is anodyne in one kind of pain, will be dolorific in another. Venesection will relieve the pain of pleurisy ; but, generally speaking, will aggravate that of spasm. Opium will do just the reverse, and so on. The following passage contains good advice.

"The exhibition of anodynes in inflammation, however, requires the exercise of much judgment. This remark appears to be the more necessary, because the employment of some of them, especially of opium and the prussic acid, has been of late strongly recommended as antiphlogistic remedies: whether they are antiphlogistic or not is not now a question for our consideration, the present object being to guard the practitioner against their indiscriminate employment in inflammation. Pain, or tenderness, is one of the most unequivocal signs of the existence of inflammation, when taken in connexion with its other symptoms; and during the treatment of inflammation, when no *anodynes* have been employed, the continuance of the pain will often afford an indication, to the practitioner, that further active measures are necessary: but it is quite certain that inflammation, in a considerable degree, may be going on while all feeling of pain is suspended by the effect of an anodyne; so that in such a case the indication of pain will be lost, the disease may be rendered obscure, and the treatment inert. Besides the reason just assigned for caution in the employment of anodynes, the stimulating quality which some of these remedies possess, ought to put the practitioner on his guard against the indiscriminate use of them when treating inflammation. It appears better, then, for the purpose of relieving pain, to depend generally on the means calculated to lessen or remove the inflammation itself, at least in all recent and active cases: this must be considered the most judicious mode of easing the feelings of the patient; but instances are daily met with in practice, especially of chronic inflammation, where the suspension of pain by anodynes is indicated by the considerations which have already been assigned." 97.

ANTHELMINTICS are treated, in a very succinct but perspicuous manner, by Dr. A. T. Thomson. He arranges the whole class under three heads—evacuant—specific—and corroborant anthelmintics. Among the evacuants, the powdered tin is placed at the head. It is supposed to act mechanically, by irritating the worms, and dislodging them from their holds on the intestines, rendering them liable to be swept away by a purgative. The dolichos pruriens acts in the same manner. The chemical anthelmintics are lime-water and alkalies, which dissolve the mucus forming the nidus of the parasitic animal. Purgative anthelmintics have no other effect than clearing away the superabundant mucus, and such worms as are detached from the coats of the intestinal canal. The specific anthelmintics are the oil of turpentine, the male fern, the bastard cabbage-tree of Jamaica, and the Indian pink, &c. Dr. Thomson has seen worms expelled by the vinum colchici, when administered for another purpose; and, therefore, thinks it entitled to the appellation of anthelmintic. The corroborants are bitters, chalybeates, and such medicines as strengthen, not only the alimentary canal, but the whole system, and thus prevent the regeneration of worms when once expelled. This article, though very concise, is very fairly executed.

ANTIPHLOGISTIC REGIMEN, by Dr. E. Barlow, occupies little more than a page of the work, and briefly adverts to the effects of bodily rest, low diet, cool air, and mental repose.

ANTISPASMODICS are disposed of by Dr. Thomson. The term is intelligent enough; but the effect is not often clear, nor the operation of the medicine satisfactory. Dr. Thomson has not been able to throw much light on the matter, as may be seen by the following short extract.

"The chief circumstance in which antispasmodics differ from narcotics is, that the administration of the former is not followed by the insensibility to impressions, and collapse, which almost invariably follows the exhibition of narcotic substances. No such effects can be induced by antispasmodics, even in large doses; yet they are as powerful as narcotics in repressing inordinate muscular action. In explaining, therefore, the difference between antispasmodics and narcotics, we may hazard the opinion that it is probable the impression exerted on the extreme nerves by a narcotic is confined to those of sensation, and must be communicated to the brain before the effect is produced; whereas that caused by an antispasmodic is confined to the nerves of motion, and produces an immediate and more permanent result by some changes effected in the state of the motor nerves, independent of any communication with the sensorium. If this opinion be correct, antispasmodics, in the strict meaning of the term, stand in the same relative position to narcotics as astringents to tonics. But, whatever may be their mode of action, the distinct nature of an antispasmodic, acting simply as such, is very obvious; and antispasmodics may be regarded as holding an intermediate place between narcotics and tonics,—less diffusible, but more durable than the former,—more immediate, but less permanent than the latter." 102.

It is hardly necessary to say, that antispasmodics comprehend every kind of stinking and odoriferous stuff in the materia medica.

Aortic Aneurism is ably treated of by Dr. Hope, who has devoted so much attention to organic diseases of the heart and large vessels. We shall introduce an extract, portraying the general signs of aneurism of the aorta.

"When an aneurism is buried deep in the chest, and not capable of being detected by the sight and touch, it does not present a single general sign which is peculiar to itself, and, therefore, pathognomonic of its existence. There are even cases in which it occasions no functional derangement—no inconvenience whatever; and the first circumstance that unveils the truth is, the sudden death of the patient, while apparently in the enjoyment of perfect health. We have met with six or seven instances in which large aneurisms had existed without awakening even a suspicion in the mind of the medical attendant. One, in particular, eluded the penetration of a distinguished foreign auscultator, though he explored the lungs with eminent success. We are acquainted with only one general sign of aneurism of the thoracic aorta which is unequivocal and certain, namely, a tumour presenting externally, and offering an expansive as well as heaving pulsation, synchronous with the action of the heart. Of the remaining general signs, a large class are identical with those of organic disease of the heart, viz. palpitation, dyspnœa, cough, tendency to syncope, terrific dreams, starting from sleep, hæmoptysis, livid or otherwise discoloured complexion, cerebral or hepatic congestions, serous infiltration, &c. This identity arises from an identity of cause; namely, an obstacle to the circulation, which depends either upon the aneurism alone, or conjointly upon it and a disease of the heart, to which, sooner or later, the aneurism almost invariably gives birth. It is obvious, therefore, that the signs of this class are equivocal. There are, however, certain other general signs which are more characteristic: yet even these are of themselves ambiguous and unsatisfactory: as they only bespeak lesions of the viscera, or derangement of their functions, but do not proclaim the latent cause of the mischief. But when they coincide with the signs derived from auscultation, they lose their ambiguity, and rise into real importance; for the two classes of signs, general and stethoscopic, are a commentary on each other, and reciprocally borrow a precision and certainty of which they are individually destitute. We shall succinctly describe the general signs to which we refer, and subjoin to each the principal sources of fallacy. The means of detecting the latter we shall point out in the final summary.

1. When the tumour has attained a considerable magnitude, the cavity of the chest is preternaturally crowded, and the patient complains of a sense of constriction, infarction, and oppression. But these sensations are common to almost all diseases of the chest.

2. The radial pulses are sometimes dissimilar, or one is extinct; an effect dependent on obstruction or obliteration of the arteria innominata, or left subclavian. But the difference of the two pulses at the wrist may proceed from a variety of causes independent of aneurism of the aorta, as, contraction of the origin of either subclavian from osseous, cartilaginous, steatomatous, or other deposition; obstructions in the course of the artery, occasioned by tumours, wounds, subclavian aneurism, &c.; an irregular subdivision of the humeral, brachial, or radial artery. We have known the most ludicrous surmises occasioned by the radial crossing to the outside at the middle of the fore-arm, and the superficialis volæ supplying its place at the wrist.

3. When the origin of either subclavian is contracted, the pulse at the corresponding wrist is a little later than the ventricular systole. We have not found this symptom uniformly present. The heart is more frequently its source than the aorta, and we have observed it to be most considerable in cases of regurgitation into the left auricle; but obstruction of the aortic valves may occasion it in a minor degree, particularly if this lesion is accompanied with extenuation or atony of the ventricular parietes. When the sign exists in both pulses, the presumption is strong that its source is in the heart.

4. According to Corvisart, a purring tremor—the *frémissement cataire* of Laennec—is sometimes perceptible to the hand at the middle or upper part of the sternum, and indicates aneurism of the ascending aorta. Purring tremor, above the clavicles, is an almost constant concomitant, and therefore a valuable sign, of dilatation of the arch; but, according to our experience, it is unfrequently and imperfectly occasioned by sacculated aneurisms, especially if lined by strata of lymph. We have never known the tremor to be occasioned below the clavicles by dilatation, unless the enlargement was so great as to extend beyond the lateral margins of the sternum, and allow the tremor to be felt through the intercostal spaces: but we have met with one case in which a dilatation of the pulmonary artery, though not voluminous, afforded a marked tremor between the cartilages of the second and third ribs on the left side: this, however, is not remarkable, as the artery *naturally* lies nearly opposite to the part described. We have never known a sacculated aneurism create a tremor below the clavicles, unless the tumour had eroded the bones of the chest, and presented externally underneath the integuments.

But the purring tremor may be occasioned in any part of the chest by mucous rattles, particularly those of the snoring kind, in the large bronchial tubes; and we have observed that, when derived from this source, it is a very common cause of deception, in reference both to aneurisms of the aorta and ossifications of the heart. Purring tremor of the pulse is regarded as a sign, though it is a fallacious one, of ossification of the aortic valves. From many dissections, it has appeared to us to be generally connected with two circumstances, viz. a powerful action of the heart, and ruggedness, without appreciable obstruction, of the aortic orifice, or interior of the vessel. It, therefore, seldom exists, unless either the action of the heart be accelerated, or the left ventricle be hypertrophous.

5. When the trachea, or primary bronchial divisions, are compressed by an aneurismal tumour, a harsh wheezing, or sibilous sound, proceeding deep from the throat, characterizes the respiration; the voice is either croaking or reduced to a whisper, or it is a compound of both; the breathing is often extremely laborious, and when the heart is simultaneously diseased, dyspnœa sometimes occurs in paroxysms of the most suffocating severity. When the œsophagus is compressed, deglutition of solids is rendered difficult, and sometimes impracticable; for the descent of the morsel excites an excruciating pain from the summit of the sternum to the spine, or lancinating deeply in every direction through the chest.

But compression of the trachea, or œsophagus, with the above symptoms, may be occasioned by tumors of any description. Wheezing respiration may proceed from an accumulation of glutinous mucus in the great branches. We have likewise known it produced in an extreme degree by laryngitis with thickening of the soft parts covering the arytænoid cartilages, and also by ossification and ulceration of the larynx from strumous, syphilitic, and mercurial disease. So difficult is it to distinguish the seat of wheezing respiration, that it has in many instances been imputed to an affection of the larynx, when it was in reality occasioned by an aneurism of the aorta; and bronchotomy has several times been actually performed with the view of obviating suffocation.

6. When the vertebræ are eroded, the patient suffers an intense terebrating pain in the spine; and when the brachial plexus of nerves is compressed by the tumour, an aching sensation pervades the left shoulder, neck, scapula, and arm, with numbness, formication, and impaired motive power of the limb. But I have met with cases in which nearly similar pains were experienced, although there was no destruction of the vertebræ; and it is common to hear individuals affected with rheumatism or spinal disease make the same complaints. The affection of the arm may be occasioned by various forms of organic disease of the heart, and it thus constitutes a part of that concatenation of symptoms which are denominated angina pectoris. We have likewise often met with it in hysterical females subject to palpitation, and occasionally in cases of pericarditis. In all these cases the pain probably originates in irritation of the cardiac plexus of the sympathetic, propagated to the brachial plexus.

7. When, in consequence of an adhesion between the aneurismal sac and the pleura, the blood plays upon the lungs, a sense of ebullition is said to be experienced. But the same symptom is familiar to individuals labouring under phthisis, or chronic mucous catarrh: and it proceeds from the successive bursting of large bubbles, formed by the transmission of air through the fluid in tuberculous caverns, or in the greater bronchial ramifications.

8. It occasionally happens that the patient suffers excruciating pain from a spasm, pursuing the course of the diaphragm, and binding the chest around, as with a cord. This symptom is too vague to be important, and it also occurs in hysteria, gastrodynia, colic, spinal diseases, and rheumatism of the diaphragm.

9. A pulsation is felt underneath the sternum, or ribs, at the superior part of the chest. This, although one of the least equivocal signs of aneurism, is not without ambiguity. It may be occasioned by a tumour of any description, as an enlarged gland, or a cancer, interposed between the sternum and the aorta, and receiving the pulsation of the latter. Even Dr. Baillie says, 'But we are not to conclude from this symptom (viz. pulsation at the superior part of the chest) that there is certainly an aneurism. I have felt the same kind of pulsation in other cases; as, for instance, where the pericardium was found strongly to adhere to the heart; where there was a slight inflammation upon the surface of the heart, with a little more water than usual in the pericardium; and where a morbid enlargement had taken place in the heart, without any aneurismal swelling.' Every one much conversant with disease must have made the same observations.

10. A pulsation is felt above the sternum or clavicles. But this may be occasioned, 1st, by enlarged glands or other tumours seated on the subclavian artery, and receiving its pulsation; 2d, by varix of the jugular vein at its junction with the subclavian; both of which conditions have deceived expert practitioners; 3d, by subclavian aneurism. This affection sometimes resembles aneurism of the aorta so exactly, that it is extremely difficult to distinguish them. Allan Burns records a case in which all the eminent surgeons of the district were unanimous in pronouncing the affection subclavian aneurism, yet it proved to be aortic.* Sir Astley Cooper has published a number of similar cases, and one is mentioned by Professor Monro tertius.† 4th. A pulsation above the sternum or clavicles may be occasioned by carotid aneurism. This, also, may readily be confounded with aneurism of the aorta, or of the subclavian artery. In April, 1826, we saw a case at Guy's Hospital, which led to much deliberation respecting the propriety of taking up the carotid above a pulsating tumour, supposed to be an aneurism of that artery. It was finally decided that the tumour was too low, and the design was judiciously abandoned. The affection proved to be a dilatation of the aorta and arteria innominata. The carotid was sound. This state of parts was indicated to us by the stethoscope. Mr. Hodgson met with a similar case.‡

11. The superior and middle parts of the chest are dull on percussion. But this sign is common to an infinity of other diseases, and the resonance is seldom impaired unless the aneurism be very large." 111.

The second part, or fasciculus, contains several important papers; but it will not be in our power to notice them all. We shall only allude to *new names*, as they appear on the long list of collaborateurs, so as to be able to introduce some specimen of contribution from each.

APHONIA AND APHTHÆ, two short articles, are from the pen of one of the most talented of contributors to this work—Dr. Archibald Robertson, of Northampton. In these two articles there is no scope for Dr. Robertson's abilities and practical knowledge; but we venture to prognosticate that these abilities will be rendered conspicuous before the Cyclopædia Medica is closed.

"We have sometimes met with aphonia depending upon atony, or relaxation of the vocal chords, in consequence of long-continued over-exertion of the voice in speaking, shouting, singing, or the like.

It is also now and then caused by ulceration of the lining membrane of the larynx and its cartilages; a disease that gives rise to rapid emaciation, hectic fever, profuse expectoration of frothy mucus, and the other frightful symptoms known to modern practitioners under the name of *phthisis laryngea*. A fatal case of this sort, most distressing in all its details, lately came under our observation, where the ulceration of the cartilages of the larynx occured as the *sequela* of syphilis.

For the most part, however, aphonia, where it occurs without any palpable disorder, or structural lesion, of the organs of speech, is a modification of hysteria; that Protean malady which assumes such various shapes and hues, and gives rise to such irregular, anomalous, and perplexing symptoms; resisting for months, or even years, the most assiduous and skilful efforts of the practitioner! In cases of this class, the loss of voice is owing to irregular distribution of the nervous influence. This again is caused by general irritability, or susceptibility, of the whole nervous system; or, in other words, by 'the hysterical temperament.' We shall seek in vain to restore the voice until we have removed that hysterical diathesis on which the loss of it depends.

*Cure.*—When the disease has not arisen from a cause which contra-indicated

* Surg. Anat. of Head and Neck, p. 30.

† Elements of Anat. vol. ii. p. 249.

‡ On the Diseases of Arteries, p. 90.

emetics, we have generally begun with one; and the following is what we have commonly employed, repeating it at intervals of three or four days, and adding to it, where the patient was robust, from gr. ss. to gr. i. of tartarised antimony:

℞. Vini Ipecacuanhæ *f* ℥ix.

Oxymellis Scillæ *f* ℥iii. M.

The success that has attended its exhibition has been most conspicuous.

Where the disease has appeared symptomatic of catarrh, we have followed up the emetic by saline, demulcent, and expectorant medicines. Leeches, but more especially blisters, to the fore part of the throat, have had an excellent effect.

Where aphonia seems premonitory of apoplexy, the most prompt and effectual depletion by blood-letting, cupping, and purgatives, must be resorted to. Where, on the other hand, it is the consequence of apoplexy, we have chiefly relied on cupping inter scapulas, leeches to the temples, a blister to the head, and, above all, a seton in the nape of the neck.

When aphonia is symptomatic of hysteria (as it so often is), the constitutional treatment adapted to the latter must be had recourse to. We would advise the following formulæ, which we have proved by experience to be well adapted to this and many other varieties of hysterical disorder:

℞. Ferri Subcarbonatis ℈i. ad ʒss.
Pulveris Valerianæ, gr. x. M. fiat pulvis ter die sumendus.

Or the following pills and mixture may be prescribed:

℞. Pilulæ Galbani Comp.

Pulveris Radicis Pyrethri āā ʒi.

Olei Anisi guttas vi. M. tere simul optime, et divide in pilulas xxiv. quarum sumat ii. vel iii. omni nocte.

Necnon, ℞. Sulphatis Ferri, gr. ii.

Acidi Sulphuric. Dil. M. x.

Solve, et adde

Infusi Gentianæ Comp. *f* ℥ix.

Aquæ Cinnamomi *f* ℥iii.

Sulphatis Magnesiæ, ʒi. M. fiat haustus bis quotidie sumendus.

With the above plan, the shower-bath every morning, at first tepid and then cold, (with or without the addition of salt to the water) may be conjoined, and should be persevered in for several weeks.

It is not unworthy of remark that, in one case of this disease, which had existed long, and resisted a great variety of remedies, we were fortunate enough to effect a cure by half-drachm doses of *balsamum copaibæ*, given three times a day, rubbed up with mucilage of gum arabic and peppermint or cinnamon water." 119.

Apoplexy is treated of by Dr. Clutterbuck—and we think the article could hardly have been put into better hands in this country. The Doctor's attention to affections of the brain and its membranes, has long been exerted, in consequence of his theory of fever, which, although it has not been productive of general conviction among the profession, must have greatly increased the author's experience and sphere of observation. The following extract will shew Dr. C.'s opinions respecting the nature, or rather the proximate cause of apoplexy. They will not be new to those who have frequented the London Medical Society, where many a long discussion has occurred on the subject.

"1st. The brain is enclosed in an unyielding case of bone, the cranium or skull, the contents of which are by this construction excluded from the influence of atmospheric pressure.

2dly. The cavity of the skull is always accurately filled by its contents, namely, the encephalon or general mass of brain, including the membranes, and vessels, and the blood contained within them. So long as the skull is perfect, there can be no rising and falling of the brain, so as in the latter case to leave a vacuity between the skull and the surface of the brain, these parts being always in actual and close contact.

3dly. The contents of the skull solid as well as fluid, if not absolutely incompressible, at least are so by any force that can by possibility be applied to them during life; and there is no air, or other elastic fluid, to be found within the cavity.

This incompressibility of the cerebral substance is easily demonstrated by experiment. It is a property by no means belonging exclusively to the brain, more than to the whole of the animal solids. This is the less to be wondered at, when it is recollected how large the proportion of water is which enters into the composition of these, and that water, as well as other fluids, is so nearly incapable of compression as to require a vast force to render it at all perceptible. But although the substance of the brain, in common with other animal solids, be incompressible, its blood-vessels will readily yield to pressure, so as to be emptied of their contents; a necessary consequence of which is, a stoppage of the circulation in the part so affected. The pressure may be made to take place on any part of the brain, even the most remote from the principal vessels; yet nevertheless the pressure, by operating through an incompressible substance, may influence vessels the most distant, so as thereby to impede, if not wholly interrupt, the cerebral circulation.

The circumstances above stated with respect to the brain, lead to very important deductions, both theoretical and practical. It follows *ex necesitate rei,* that no material variation can take place, within a short period, in regard to the absolute quantity of blood in the brain. No additional quantity can be admitted into the blood-vessels situated there, the cavity of the skull being already completely filled by its contents. A plethoric state or over-fulness of the cerebral vessels altogether, though often talked of, can have no real existence; nor on the other hand can the quantity of blood within the vessels of the brain be diminished, any more than can wine or other fluid be drawn from a cask without furnishing an equivalent for the portion abstracted from it, by the supply of an equal bulk of air, which in the case of the brain can of course find no entrance. No abstraction of blood therefore, whether it be from the arm or other part of the general system, or from the jugular veins, (and still less from the temporal arteries,) can have any effect on the blood-vessels of the brain, so as to lessen the absolute quantity of blood contained within them.

From the experiments of Dr. Kellie, it was found that in animals bled to death the brain still contained the usual quantity of blood; and in some cases the superficial veins were found gorged with blood, and the sinuses full; the rest of the body being at the same time blanched, and drained of its blood. In a few instances, the brain appeared to contain less blood than usual; but then there was found some serous exudation. When the cranium of the animals subjected to these experiments was perforated before they were bled, the brain was as much emptied of its blood as the rest of the body. In two instances of persons that had been hanged, the cellular membrane of the whole head externally was turgid with blood; but nothing peculiar was observed in the state of the vessels of the brain itself. When blood is suddenly or rapidly extravasated any where within the skull, the space thus occupied can only be furnished by the compression and consequent emptying of the blood-vessels in other parts of the brain; and in the same degree that this happens, it is evident that the circulation of such parts must be interrupted. But in the formation of tumours within the skull, and during the slow accumulation of serum from inflammation or any other cause, the cerebral substance itself may be absorbed, to an extent corresponding with the bulk of the tumour, or the quantity of serum deposited. The circulation of the brain may then go on uninterruptedly, and thus the apoplectic symptoms be prevented.

But although under ordinary circumstances, the absolute quantity of blood contained within the vessels of the brain must remain the same, there may be great differences in regard to its distribution, and the force and velocity with which it is moved. Thus, the arteries of the brain altogether may be unusually distended with blood; but in this case the veins will be in the same degree compressed and emptied, and the circulation of the organ proportionally interrupted, with a corresponding interruption of functions.

Again, there may be a partial fulness or distension of vessels in one part of the brain only, but this must be at the expense of the rest of the brain, which will be proportionally deprived of the usual supply of blood. This will be the case in circumscribed cerebral inflammation, as well as in other cases of partial excitement of the organ. The different parts of the brain will then be in different, and perhaps opposite states, in regard to the performance of their functions. The functions of one of the cerebral organs may be excited, while those of another may be imperfectly carried on or depressed. Such an inequality in the state of the cerebral functions is observable in most diseases of the brain; for there are probably few in which the whole organ is simultaneously and equally affected.

In like manner there may be great diversity with respect to the force and velocity of circulation in the brain; the absolute quantity of blood in the vessels remaining still the same. In this way the functions may be more or less excited, or more or less disturbed. These changes in the state of the cerebral circulation are all independent of the heart, the action of which has but little influence over the brain or its functions. It follows, from what has been now stated, that blood-letting, when employed as a remedy in apoplexy, or other brain-affections, however useful it may be and undoubtedly is, in many cases, does not effect its purpose by diminishing in any degree the absolute quantity of blood in the brain, but by reducing the velocity and impetus of the circulation there, and which it does by influencing the general system.

We have seen that the changes observed in the brain of apoplectic persons are very dissimilar; and that, in some instances, as has been stated above, no material deviation from the natural state of parts has been found; it is plain, therefore, that such changes, when they do occur, cannot be the proximate cause, the *causa continens*, of the symptoms, but are to be considered, at most, as remote causes; and, with regard to some of them, are to be looked upon rather as mere coincidences than causes.

The opinion that appears to prevail most generally at present, as to the immediate cause of the suspension of functions that constitutes the apoplectic state, is that the remote causes of the disease, such as extravasated blood, and accumulation of serum, produce a compression of the cerebral substance, thereby interrupting its functions. But, besides that some of the remote causes of apoplexy have no apparent tendency to make any direct pressure on the brain, it must not be overlooked that the cerebral substance being in its nature incompressible, cannot, so long as the blood is contained within its vessels, be exposed to greater pressure at one time than another. It must be in some other way, therefore, than by compression of the substance of the brain, that the remote causes act in producing their effect. It cannot be questioned that pressure on the brain of any kind, if carried to a certain extent, is capable of interrupting the functions of the organ so as to induce apoplexy; but there is good reason to believe that the pressure operates upon the blood-vessels, so as to impede mechanically the passage of the blood through them;—in a word, that interrupted circulation in the brain is the proximate or immediate cause of that temporary suspension of the sensorial functions which constitutes the apoplectic state."

The Doctor's peculiar views are, that the veins of the brain are never over-distended during life—and that the congestion or distention is always in the arterial system. We need hardly say that he is unable to shew good proof of this theory. We are ready to grant that, from the peculiar condition in which the brain is placed, there can be very little additional blood forced into it at any time; but the *pressure* upon the organ may be very much more at one time than another—and, from disease of vessels or parts, there may be much *inequality* of the circulation induced by apoplectic causes.

Dr. C. makes some judicious observations on the profuse and indiscriminate venesections which are performed in apoplexy, without regard to the exciting causes, or to the true nature of the disease. His observations will be productive of good effects in many cases—they will puzzle the inexperienced practitioner in others.

PULMONARY APOPLEXY. This is shortly but satisfactorily portrayed by Dr. Townsend, well known as the translator of Andral. As this is an affection comparatively little known to the generality of medical men in this country, we shall endeavour, as briefly as possible, to put our readers in possession of the present stock of information on the subject. The name is obviously improper; but, having been once introduced and established, we suppose it will now be retained. Andral has proposed to denominate the disease *pneumo-hæmorrhagia,* in opposition to *broncho-hæmorrhagia,* in which the blood escapes from the mucous membrane of the bronchi. In pulmonary apoplexy, blood is extravasated into the vesicular, and probably also into the cellular membrane of the lung. Now hæmoptysis may arise either from bronchial hæmorrhage or exhalation, when no rupture of vessels can be discerned with the greatest care after death; or from rupture of vessels and extravasation into the substance of the lung, that is, from pulmonary apoplexy. The former cause is infinitely the more frequent. The disease was hinted at by Haller—described by M. Lereille in 1816—by Dr. Hohntaum, under its present name, in 1817, and best of all by M. Laennec, in 1819. It varies in its symptoms, causes, degrees, anatomical characters. It may be broadly said to have two leading forms, the circumscribed and the diffused. M. Laennec's description of the anatomical characters of the former cannot be surpassed in accuracy.

"A remarkable induration of the pulmonary substance, equal to that of the completest hepatization: the induration, however, is very different from the inflammatory affection of the lungs distinguished by this term. It is always partial, and rarely ever occupies a considerable portion of the lungs: its more ordinary extent being from one to four cubic inches. It is almost always very exactly circumscribed, the induration being as considerable at the very point of termination as in the centre. The pulmonary tissue around is quite sound and crepitous, and has no appearance whatever of that progressive induration found in pneumonia. The substance of the lung is indeed often very pale around the hæmoptysical induration; sometimes, however, it is rose-coloured, or even red, as if tinged with fresh blood; but even in this case the circumscription of the indurated part is equally distinct. The indurated portion is of a very dark red, exactly like that of a clot of venous blood. When cut into, the surface of the incisions is granulated as in a hepatized lung; but in their other characters these two kinds of pulmonic induration are entirely different. In the second degree of hepatization, we can perceive distinctly the black pulmonary spots, the blood-vessels, and the fine cellular intersectures, all of which together give to this morbid state the aspect of certain kinds of granite. In the induration of hæmoptysis, on the contrary, the diseased part appears quite homogeneous, being altogether black or of a very deep brown, and disclosing nothing of the natural texture of the part, except the bronchial tubes and the larger blood-vessels. The latter have even lost their natural colour, and are stained with blood. In scraping the incised surfaces of their parts, we can detach a small portion of very dark, half-congealed blood, but in a much less proportion than we can press out the bloody serum from a hepatized lung. We sometimes find two or three similar indurations in the same lung, and frequently both lungs are affected at the same time."—*Laennec, translated by Forbes.*"

To verify the above description, the extravasations of blood must be limited in extent, circumscribed by the interlobular septa, and the patient must have survived, as he usually does, for a certain length of time, in order that coagulation and condensation of the fluid blood may have taken place. In the other form of the malady, which is still but a variation in degree, not in kind, the extravasation is greater, the blood diffused through the texture of the lung, which is broken down by it, and the former may even have burst through the pleura pulmonalis and lie in the pleural cavity. There is also a sort of intermediate grade between the two, in which the centre of a mass of hæmoptoic engorgement is soft, and filled with a clot of pure blood. A young man died of organic disease of the heart, in the Whitworth Chronic Hospital, after experiencing a violent attack of hæmoptysis about a week before his death. The lower lobe of the right lung was occupied by a mass of hæmoptoic engorgement as large as an orange, and contained in its centre a clot of dark-coloured blood as large as a hazel-nut, the cavity having been evidently formed at the expense of the pulmonary parenchyma.

In the more advanced, or rather more severe variety, for it is a measure of extent, and not of stages, the extravasation is not circumscribed, solid, nor granular when incised, but soft and fluctuating to the touch, and, when cut into, exhibiting a mixture of fluid and clotted blood, diffused through the parenchyma of the lung, which is ruptured and broken down. The blood, in these cases, is seldom coagulated, for which circumstance several reasons may be assigned. The hæmorrhage is generally so violent that it supervenes speedily, and these extensive hæmorrhages are commonly connected with a fluid state of the blood, diminishing its tendency to coagulation, and disposing it to pass off more freely by the exhalants, as in scurvy, purpura, &c. The mass, too, of the extravasation may render coagulation less active than in the circumscribed effusions. However, this of itself would probably signify but little, as, in the large extravasations following injuries of the lung, we find more or less coagulation take place readily enough.

As an example of this form of pulmonary apoplexy, we may mention a case which occurred under the notice of Dr. Townsend, in the Hardwicke Fever Hospital. A young, delicate-looking man, in an advanced stage of fever, stooping out of bed to take up his spitting-pot, lost all consciousness and fell on the floor. He remained insensible for some minutes, threw up a large quantity of blood, then recovered so far as to ask for water, which he drank with avidity, again relapsed into apparent insensibility, and died within an hour, after discharging a quantity of blood from his mouth and nose. On dissection, eighteen hours after death, all the air-passages, from the mouth to the lungs, were filled with dark fluid blood. The middle and lower lobes of the right lung were externally of an uniform deep red colour, and, when pressed under the fingers, conveyed a distinct sense of fluctuation. When cut into, a quantity of fluid blood rushed out, with grumous clots, and several masses of broken-down pulmonary tissue. The interior of the lung presented a shreddy appearance, and resembled sponge, steeped to saturation in blood.

Dr. Townsend has only been able to discover four recorded cases, in which the hæmorrhage burst through the pulmonary pleura. The first is related by Corvisart *(Commentaires sur la Traité de la Percussion)*; the second by M. Bagh, in the Révue Médicale; the third by Andral *(Clinique Médicale)*; the fourth, being the most circumstantial, we shall extract in Dr. Townsend's words.

"The fourth and last case of this description on record is that recently published by Dr. J. C Ferguson, in the first volume of the Dublin Medical Transactions. A robust man, æt. 36, who had occasionally suffered from attacks of constipation and bronchitis, complained, on the 19th of June, 1822, that his cough was increased, his chest somewhat oppressed, and his expectoration, since the day preceding, slightly tinged with blood; his countenance was pale, his pulse about 90, and feeble, and his skin covered with a cold clammy perspiration. On the next day he felt relieved by the operation of a purgative. During the succeeding night he was rather restless; however he ate his breakfast as usual, and while in the act of stooping to put on his shoes, he complained to his wife of loss of vision, seemed to faint, and died without a struggle. Examination made forty-eight hours after death:—the left pleural sac contained about three quarts of blood, the serum supernatant, as in blood allowed to stand after venesection, and the clot in considerable quantity, but very soft, occupying the most dependent portion of the cavity. The lung had contracted no adhesion; the superior lobe was one mass of the most perfect pulmonary apoplexy, the structure of the lung seeming to be actually broken up by the excessive effusion of blood into it; the apoplectic mass was soft and flabby; it would scarcely bear to be incised, but broke down easily under the finger or scalpel. On the superior and posterior part of the affected lobe a laceration of the investing pleura was found, about one inch in length and half an inch in breadth, with very irregular edges, and immediately over the point where the sanguineous effusion into the substance of the lung seemed most intense, and where we might naturally expect the greatest violence to be opposed to its serous covering. A remarkable fact in the history of this case was, that he had expectorated no blood for fourteen hours before death, nor in the agony was there any escape of blood from the mouth or nares which might lead to a suspicion of the real seat of the disease. The same remark is also applicable to the preceding cases, Nos. 1 and 2, in which no hæmoptysis whatever took place before death, and the cause of the fatal catastrophe was only discovered on dissection." 137.

The etiology of the disease is rather conjectural than positive; we must pass it by. The study of the causes is of more urgent importance. The occasional causes are in general the same as those of hæmoptysis, suppression of discharges, plethora, &c. But ordinary hæmoptysis is more frequently dependent on tubercles, pulmonary apoplexy on organic disease of the heart, a distinction of paramount importance. Of twenty-two cases of pulmonary apoplexy examined by Dr. Townsend after death, fifteen were those of individuals labouring under disease of the heart; two were connected with tubercles of the lungs; one with external injury; and, in four, no organic disease of heart or lung, nor other obvious cause, was discovered. We might enumerate many authors who have pointed out the connexion between pulmonary apoplexy and cardiac disease, but the fact is sufficiently certain.

The remarks made by Bertin are so reasonable in themselves, and square so well with what we have personally witnessed, that we cannot refrain from quoting Dr. T.'s short account of them.

"According to this author, hypertrophy of the right ventricle has the same tendency to produce apoplexy of the lung, that hypertrophy of the left has to cause apoplexy of the brain, and by the same mechanism; for as the brain directly receives the shock of the column of blood which is propelled by the aorta through the left ventricle, so the lungs receive directly the shock of blood which is propelled through the pulmonary artery by the right ventricle. Accordingly, when the parietes of the right ventricle acquire an increased volume and proportionate increase of energy in their contractions, the blood is propelled through the pulmonary vessels with such an increased degree of force as is sometimes sufficient to over-distend and rupture their parietes. The hemorrhage produced in this way he considers to be of an active character, and essentially different from the passive hemorrhage which results from the over-distention and rupture of the pulmonary capillaries, arising from the mechanical congestion of the lung caused by narrowing of the left auriculo-ventricular orifice. Of all diseases of the heart, these have the strongest tendency to produce attacks of pulmonary apoplexy, in consequence of the direct influence they exert over the pulmonary circulation; but the same effect may likewise be produced by any disease of this organ which obstructs the free transmission of the blood." 138.

We have seen six or seven cases of decided pulmonary apoplexy, and several other doubtful ones. Of the former the majority, five we think, were connected with disease of the heart, and more than that with a particular form of such disease. There was contraction of the left auriculo-ventricular orifice, dilatation of the left auricle, and pulmonary vessels, and hypertrophy with dilatation of the right ventricle. Under such circumstances, the occurrence of pulmonary apoplexy is neither surprising nor difficult of explanation; the rationale indeed is obvious. In one of M. Cruveilhier's cases the same complication existed, and M. Bertin's observations bear directly on the point. Our own cases, at least several of them, have been published in a former number of this Journal, and on the whole we do not think that Dr. Townsend has drawn so much attention as he might and should have done, to the connexion between pulmonary apoplexy and contraction of the left auriculo-ventricular opening of the heart. Dr. T. has seen two cases of pulmonary apoplexy with hypertrophy of the left and passive dilatation of the right ventricle. Many cases of the disease are met with, where undoubtedly none of the complications and causes above-mentioned are discoverable, but these are the minority, and it will be found that they are mostly of the diffuse species; that is, where sudden and violent effusions of blood occur in the lung, from what we may consider as in some measure accidental circumstances. The circumscribed extravasations, as they are of longer continuance, and frequently display the signs of different degrees of age, so they also evince a more permanent, organic, and constantly acting cause. These, at least, are the conclusions to which the facts seem to us to lead, although we should be far from dogmatising on the subject; there is much yet to be learned.

"The *diagnosis* of pulmonary apoplexy is, in many cases, a matter of considerable difficulty. When the patient dies suddenly without hæmoptysis, as in cases No. 1 and 2 already recorded, it will often be impossible to determine, except by inspection, whether death was caused by cerebral or pulmonary apoplexy, or by rupture of the heart: and even in those cases where the fatal attack is accompanied with hæmoptysis, the physician will sometimes find considerable difficulty in ascertaining the source of the hæmorrhage, as the bursting of an aneurismal sac into the bronchia may produce effects precisely similar. In the more common and less violent forms of the disease, in which an accurate diagnosis is of much more practical importance, the symptoms most pathognomonic, as enumerated by Laennec, are, violent sense of oppression in the chest; great difficulty of breathing; cough, accompanied with irritation of the larynx, and sometimes by very acute pain of the chest; expectoration of bright and frothy, or black and clotted blood, quite pure, or mixed with saliva or mucus; frequent full pulse, with a particular kind of vibration even when soft or weak, as it frequently is after a day or two. There is rarely any positive fever, and the heat of the skin continues natural, or nearly so; frequently the heart and arteries yield the bellows sound to a very marked degree. Of all these symptoms the spitting of blood is the most constant and most severe, and returns by fits, accompanied with cough, oppression, anxiety, intense redness or extreme paleness of the face, and coldness of the extremities. When the hemorrhage is very great, it comes on sometimes with a very moderate degree of cough, accompanied by a convulsive elevation of the diaphragm, like that which takes place in vomiting." 139.

But of these symptoms none are constantly present, and none are therefore pathognomonic. Hæmoptysis certainly is not, for of the cases which we witnessed, it was not distinctly present in any, and the remark is a general one. Laennec observes that the slighter cases of hæmoptysis depend on a simple exhalation from the bronchia, whilst those of violent and extreme hæmorrhage almost invariably proceed from the vesicular structure of the lung. This must be taken with reservation, and it would require a careful survey of the particulars of all the cases known, to make any sound or fair calculation of the value of hæmoptysis as an evidence of the existence of pulmonary apoplexy. But do physical signs afford any certain indications? We know from experience that they do not, and although they may assist in forming a judgment or confirming one, they are by no means adequate of themselves to lead to certain diagnosis. Auscultation has suffered more from its friends than from its enemies. The physical signs are as follow:—

"These signs are dulness of sound in that part of the chest which corresponds with the seat of the disease, and the total absence of all respiratory murmur in the same circumscribed space, together with a crepitating râle around this space; this râle which here indicates a slight infiltration of blood, is always found at the commencement of the disease, but is frequently wanting in the latter stages. When these signs co-exist with pulmonary hemorrhage, we may be assured that the seat of the hemorrhage is in the substance of the lung, and not simply in the bronchia. Besides these, which may be considered as the pathognomonic signs of pulmonary apoplexy, there is likewise, especially at the root of the lungs where the larger bronchia are situated, a mucous râle with bubbles, which seem to be large and thin, and formed by a matter more liquid than mucus; they also burst more frequently, and with a peculiarity of sound which cannot be mistaken. As the most common seat of this disease is in the central parts of the lower lobe, or towards the middle and posterior part of the lungs, it is consequently on the posterior and inferior part of the chest, that we ought to search for them with the stethoscope." 139.

The large extravasations are more likely to be recognized than the small, if the patient's demise is not too rapid to allow of accurate examination. The danger to be apprehended obviously depends on the causes of the hemorrhage, the quantity, and the patient's previous condition. On these points we need not dwell. Laennec regards the resolution of hæmoptoic engorgement as taking place with facility, whatever may be its severity, and describes the anatomical characters of the extravasations in their progress to this state. M. Bouillaud relates a case, in which a clot of pulmonary apoplexy was seen surrounded by a well-organized cyst. Andral conceives that the clot may become organized, and form a nidus for different morbid productions, tubercle, melanosis, pus, &c. This is rather speculation than proven fact, and so we leave it. It is more consistent with what is often seen and satisfactorily known, to believe that pulmonary apoplexy may lead to the death of the part, which is choked, as it were, with blood, and to its consequent sloughing. It is sufficient, however, to point out this result.

On the treatment of the disease we need say little. It is commonly but a symptom, at all events a consequence or concomitant, of other diseases of a serious character. As a symptom, it must be treated like hæmoptysis, with more caution, perhaps, but on the same principles. As an attendant on other diseases, those morbid states or actions must be distinguished first, and then encountered on the principles and practice which always guide us. Further than this, we really conceive that nothing need be said. To enter on the special treatment of pulmonary apoplexy, would be to enter on the treatment of hæmoptysis, which is foreign to our purpose. We all know the powers of general bleeding in the latter.

The Diseases of Artisans, by Dr. Darwall, are very fully considered, and the Doctor, besides his local knowledge of the subject, has consulted the best authorities. We are unable to do more than allude to and commend the article.

Ascites is by the same author: and is not much developed. But abdominal dropsy is so rarely an original disease, that its consideration comes more properly under other heads.

We have already made extracts from Dr. Roget, who writes the article Asphyxia with his usual talent.

Asthma and Auscultation are by Dr. Forbes, and are, as usual, much more extended, proportionally, than the other subjects. Either Dr. F. should condense, or his collaborateurs should dilate, else the writings of one author will weigh down a dozen of the others. It is difficult, or perhaps impossible, to adjust these things with much nicety; but we suspect that our talented friend, Dr. Forbes, is rather small in the organ of *concentrativeness*. His matter is always good—but many words might be spared without loss. In no kind of work can diffuseness be so well spared as in a Cyclopædia of Medicine. The following extract is interesting.

"There are several forms of spasmodic stricture of the upper portion of the air-tube, more particularly of the glottis, well known to practical physicians. The more common of these occur in infants and young children; but they are confined to no particular period of life. An old lady, a friend of the writer of this article, has all her life been liable to attacks of this kind, which seize her instantaneously, after long intervals, and, during their continuance, entirely prevent inspiration, and thus threaten immediate death. After a few seconds the paroxysm subsides, without leaving a trace behind it. The disease described by Dr. John Clarke as a 'peculiar species of convulsion in infant children,' and of which a more complete account has recently been published by Dr. Marsh,* is evidently a spasmodic affection of the same kind, but less strictly confined to the glottis than in the case just mentioned. In the infantile disorder, the spasmodic disposition frequently extends over the whole muscular system, inducing general convulsions. The disease frequently termed *spasmodic croup*, and which occurs also most commonly in children, but at a somewhat later period, consists, in like manner, in numerous instances, in a spasmodic affection of the glottis; but, in this case likewise, the spasms frequently extend further, in many instances affecting the greater part of the air-tubes. When this is the case, the disease is in truth a form of asthma, and ought rather to be named infantile asthma than spasmodic croup. A few practical writers represent the common form of asthma, in adults, as consisting almost exclusively of spasm of the same parts; and, among others, Dr. Wilson Philip. 'The spasmodic asthma (says this author, in his recent work,) appears, properly speaking, not to be a disease of the lungs themselves, but of the parts of the upper extremity of the windpipe, in which the contraction of the passage of the air, by spasm of its muscles, produces the violent struggling for breath which attends its paroxysms.' We shall examine the validity of this opinion after having noticed the other theories. The chief of these is that which represents the asthmatic paroxysm as consisting in a spasm of the external muscles of respiration.

One would imagine that it could be no very difficult matter to determine, after an inspection of the naked chest of a patient in a fit of asthma, whether the external respiratory muscles were in a state of spasm or not: and we cannot help suspecting that some of those who have been the strongest advocates for the affirmative of this proposition, have taken but little trouble to ascertain its truth in the most natural and simple way. Our own observations have invariably led to the conclusion that all the muscles of which the action is perceptible, are, during the paroxysm, exerting themselves strongly to *dilate* the chest; and there are the strongest reasons for believing that the muscles, the action of which is concealed, are in the same condition. It is universally admitted that expiration is, in ordinary cases, effected, in a great measure, by the mere mechanical sinking of the walls of the chest, upon the cessation of the active muscular efforts of inspiration. The ribs descend and the diaphragm ascends, and necessarily, upon the relaxation of the muscular fibres, the contraction of which had enlarged the cavity of the chest. And if we take into consideration the immense superiority of power of the muscles of inspiration, and remember that these are all, during the paroxysm, exerting themselves to the utmost to dilate the chest, it seems extremely improbable that the power of their feeble antagonists, however exerted, could resist them.

But independently of these negative proofs, we have many direct ones that the principal site of the spasm is in the air-passages, and that the instinctive and voluntary efforts of the muscles of inspiration are almost exclusively directed to overcome this. Some of these direct proofs are the following. Asthma, as we shall soon see more particularly, is very commonly dependent on a diseased state of the bronchial membrane, and the attacks are frequently induced by an increase of this state. The resolution of a paroxysm is almost invariably accompanied by a great increase of secretion from the bronchi, which could hardly be expected to be the case if the affection were seated either in the muscles of the glottis, or in the *external* muscles of respiration. Diseases of the bronchi, which produce a similar contraction of the air-passages by mere swelling of the membrane, namely bronchitis, and an affection producing nearly the same physical

* Dublin Hosp. Reports, vol. v. p. 600.

condition of the parts from a sudden congestion of the blood-vessels, give rise to a state of respiration very like that which obtains in the asthmatic paroxysm. Auscultation of the respiration, during the paroxysm, discovers particular sounds over the whole extent of the chest, which sounds can only have their site in the bronchial tubes; and as these sounds come on and go off with the paroxysm, it seems a necessary conclusion that they depend on the physical changes produced by this, and that those changes take place in the bronchi. Common wheezing, as heard by the unassisted ear in asthma and in most cases of dyspnœa, may exist in a very marked degree without the auscultatory rhonchus, which may be considered the pathognomonic sign of bronchial infarction from whatever cause derived.

But while, for these reasons, we think it must be allowed that the main site of the spasms which constitute the asthmatic paroxysm is the bronchi, we are by no means of opinion that they are exclusively confined to these. On the contrary, we think it very certain that not only all the parts already mentioned, but others still more remote, are frequently involved in the same disordered action. This is what must have been anticipated from a consideration of the very nature of the affection. In all spasmodic diseases there is a disposition towards extension of the spasm from the original or principal site, and, indeed, this fact is perhaps more easily explained on physiological principles than the restriction of the spasm to one part or one set of muscles could be. In all these cases, if the nervous centres are not primarily affected, they invariably become so subsequently; and the circumscription of the local manifestation of the spasm to the muscles first disordered thence becomes very improbable. Besides, we know that muscles whose actions are at all associated, are very liable to suffer generally when one or more of the class are morbidly affected. And, in the present case, we admit that the muscles both of the larynx and the chest are frequently involved in the progress of the paroxysm. They are, however, we believe, in almost every case, affected secondarily, and it is therefore impossible to subscribe to the opinions of those who wish to make them the chief site of the asthmatic spasm. Other muscular affections, of a spasmodic kind, are not uncommon in the paroxysm of asthma. The sudden calls to empty the bowels are no doubt partly owing to a morbid action of the muscles concerned in this process, although chiefly, perhaps, the consequence of the same general disorder of the nervous and vascular systems, which gives rise to the pallid and shrunk state of the surface, the flow of limpid urine, &c. In a young man, now under our care, subject for some years to periodic asthma, which generally attacks him during sleep, the first indication he has of the invasion of the paroxysm, is an involuntary spasmodic twitching of the right foot or right leg, of which he is conscious for some time before he is fully awake, and of which the increasing violence awakens him. The twitching always leaves him on his becoming fully awake, but he then finds himself under the full dominion of his asthma.

It must not be imagined from any thing that has been stated, that we consider the asthmatic paroxysm as consisting exclusively of a muscular spasm of parts otherwise healthy. This, indeed, may be the case in a few instances; but it is not to be doubted that, in the great majority of cases, the spasm not merely affects parts previously diseased, but that the phenomena of the paroxysm are partly dependent on, and greatly modified by, these very lesions co-existing with the spasm, aggravating it, and, in turn, being aggravated by it." 188.

We must now close our notice of the first two numbers of this work. We have little doubt that the Cyclopædia of Practical Medicine will prove to be far superior to any thing of the kind which has yet appeared on this side of the Channel, though it may not equal some of the dictionaries of medicine in France. The reason is obvious. In France, the very first practitioners engage in such undertakings. In England, the prime actors in the medical drama are to much engaged in making money to attend to any literary concern in the way of joint-stock company. But, though the greatest names may not appear on the title-page, the work may be better than if they did.

